# Prediction of Glass Transition Temperature of Polymers Using Simple Machine Learning

**DOI:** 10.3390/polym16172464

**Published:** 2024-08-29

**Authors:** Jaka Fajar Fatriansyah, Baiq Diffa Pakarti Linuwih, Yossi Andreano, Intan Septia Sari, Andreas Federico, Muhammad Anis, Siti Norasmah Surip, Mariatti Jaafar

**Affiliations:** 1 Department of Metallurgical and Materials Engineering, Faculty of Engineering, Universitas Indonesia, Kampus UI Depok, Depok 16424, Indonesiayossiandreano06@gmail.com (Y.A.); intn.septs@gmail.com (I.S.S.); andreas.federico@ui.ac.id (A.F.);; 2 Advanced Functional Material Research Group, Faculty of Engineering, Universitas Indonesia, Kampus UI Depok, Depok 16424, Indonesia; 3 Faculty of Applied Sciences, Universiti Teknologi MARA, Shah Alam 40450, Malaysia; snorasmah@uitm.edu.my; 4 School of Materials and Mineral Resources Engineering, Universiti Sains Malaysia (USM), Nibong Tebal 14300, Malaysia; mariatti@usm.my

**Keywords:** polymer, glass transition temperature, SMILES, machine learning, deep learning

## Abstract

Polymer materials have garnered significant attention due to their exceptional mechanical properties and diverse industrial applications. Understanding the glass transition temperature (*T_g_*) of polymers is critical to prevent operational failures at specific temperatures. Traditional methods for measuring *T_g_*, such as differential scanning calorimetry (DSC) and dynamic mechanical analysis, while accurate, are often time-consuming, costly, and susceptible to inaccuracies due to random and uncertain factors. To address these limitations, the aim of the present study is to investigate the potential of Simplified Molecular Input Line Entry System (SMILES) as descriptors in simple machine learning models to predict *T_g_* efficiently and reliably. Five models were utilized: k-nearest neighbors (KNNs), support vector regression (SVR), extreme gradient boosting (XGBoost), artificial neural network (ANN), and recurrent neural network (RNN). SMILES descriptors were converted into numerical data using either One Hot Encoding (OHE) or Natural Language Processing (NLP). The study found that SMILES inputs with fewer than 200 characters were inadequate for accurately describing compound structures, while inputs exceeding 200 characters diminished model performance due to the curse of dimensionality. The ANN model achieved the highest R^2^ value of 0.79; however, the XGB model, with an R^2^ value of 0.774, exhibited the highest stability and shorter training times compared to other models, making it the preferred choice for *T_g_* prediction. The efficiency of the OHE method over NLP was demonstrated by faster training times across the KNN, SVR, XGB, and ANN models. Validation of new polymer data showed the XGB model’s robustness, with an average prediction deviation of 9.76 from actual *T_g_* values. These findings underscore the importance of optimizing SMILES conversion methods and model parameters to enhance prediction reliability. Future research should focus on improving model accuracy and generalizability by incorporating additional features and advanced techniques. This study contributes to the development of efficient and reliable predictive models for polymer properties, facilitating the design and application of new polymer materials.

## 1. Introduction

Polymer materials have long been a focus of research due to their superior mechanical properties and wide range of applications across various industries. Polymers offer numerous advantages, including resistance to corrosion, ease of molding, low density, and specific gravity, making them efficient for installation and transportation [1,2,3]. In their applications, a deep understanding of polymer properties, particularly the glass transition temperature (*T_g_*), is crucial to avoid operational failures at certain temperatures [4,5].

Traditionally, *T_g_* testing is conducted using methods such as trial and error, dilatometry, differential scanning calorimetry (DSC), and dynamic mechanical analysis [6,7,8]. Although these methods can provide reasonably accurate results, they tend to be time-consuming and costly, and often exhibit unstable accuracy [9]. Conventional testing also faces challenges in managing random and uncertain factors affecting polymer properties, which can lead to erroneous data [9]. Therefore, there is a need for more efficient and reliable methods to predict polymer *T_g_*.

In response to these challenges, simulation methods emerged as a significant step forward [10,11]. Simulations, such as molecular dynamics (MD) and Monte Carlo (MC) simulations, provided a way to predict polymer behavior and properties by modeling the interactions at the molecular level. These approaches have been valuable in understanding polymer physics and predicting *T_g_* with greater control over experimental variables. However, they also come with limitations, such as high computational costs and the need for extensive expertise to interpret the results accurately.

In the ongoing quest for efficiency and accuracy, the advent of machine learning has introduced a transformative approach to polymer research. One emerging paradigm for addressing the limitations of conventional testing and simulations is machine learning. Previous research has attempted to leverage machine learning and deep learning to predict polymer properties. Zhang et al. [12] used deep learning models to predict the mechanical properties of polymers, while Liu et al. [10] employed artificial neural networks (ANN) to predict *T_g_* based on density functional theory and quantitative structure-property relationships (QSPRs). However, these studies still face limitations in terms of limited data and complex model interpretation. Other research, such as that conducted by Guang Chen et al. [13] using recurrent neural networks (RNNs) and Chan et al. [14] using convolutional neural networks (CNNs), also showed limitations in data representation and the influence of molecular structure.

This study offers a solution by using simple machine learning, represented by k-nearest neighbors (KNNs), support vector regression (SVR), extreme gradient boosting (XGBoost), and deep learning, represented by ANN and RNN, to predict the *T_g_* more accurately and efficiently. By utilizing the Simplified Molecular Input Line Entry System (SMILES) to represent molecular structures, this study aims to develop a better *T_g_* prediction system. These two approaches were compared to determine the method that provides prediction performance, with the hope of making a significant contribution to the efficiency of polymer material research and applications.

## 2. Materials and Methods

### 2.1. Data Collection and Preparation

This study collected *T_g_* data, and monomer structures represented SMILES from the PolyInfo database available on the MatNavi NIMS website. The polymers used in this study are homopolymers with simple molecular structures consisting of only one type of monomer. After the collection and cleaning process, 1437 data points were obtained and prepared as the dataset. Before being used to train the model, the SMILES descriptors needed to be converted into numerical data using either Natural Language Processing (NLP) or One Hot Encoding (OHE) methods. The NLP method employs char embedding, which transforms characters in SMILES into numerical forms based on their positions in the character lexicon. In contrast, the One Hot Encoding process uses molecular fingerprints generated with the RDKit library to produce binary vectors. Descriptive statistics of the number of SMILES characters and *T_g_* can be seen in Table 1.

### 2.2. Machine Learning Modeling

This study utilized three simple machine learning models and two deep machine learning models built using the programming language Python version 3.11.3 and the Visual Studio Code platform version 1.92.0 to predict the *T_g_* of polymers; these models consist of k-nearest neighbors (KNNs), support vector regression (SVR), extreme gradient boosting (XGB), which represents simple machine learning and artificial neural network (ANN), and recurrent neural network (RNN), which represents deep machine learning. The RNN model used SMILES converted into numerical data via the Natural Language Processing (NLP) method as input for predicting the *T_g_*. Meanwhile, the KNN, SVR, XGBoost, and ANN models were trained using the OHE method to transform the SMILES into binary vectors as input.

The KNN, SVR, and XGBoost models are representations of simple machine learning methods. KNN is a non-parametric supervised learning technique that uses the k-nearest training samples in a dataset as input to predict the property value for a given object. The given value represents the average of the k-nearest neighbors’ values. If k equals 1, the nearest neighbor receives the output directly. KNN has numerous key advantages, such as simplicity, efficacy, intuitiveness, and strong classification performance across several domains, and it exhibits resilience to noisy training data and demonstrates effectiveness when the training data is extensive. The k-nearest neighbor (KNN) algorithm may exhibit suboptimal computational efficiency when dealing with a sizable training dataset. The model is very responsive to extraneous or repetitive characteristics, as all characteristics contribute to the similarity and hence to the categorization [15]. The KNN model utilizes the parameter n_neighbors, which represents the number of nearest data points used to predict the output value of an input sample.

Support vector regression (SVR) is a regression technique that uses support vectors to identify a hyperplane that minimizes error within a certain margin. This approach is very effective at handling outliers, making it robust. However, if the relationship between input and output becomes complex, overfitting may occur. The SVR model employs the parameters kernel, C, and gamma [16]. The kernel parameter is a function used to map data from the original input space to a higher-dimensional feature space, allowing data that is not linearly separable in the original input space to become linearly separable in the feature space. The gamma parameter determines the curvature of the decision boundary the model creates, while the C parameter adjusts the trade-off between margin size and the error it generates [17].

The XGBoost model is an ensemble learning-based machine learning algorithm composed of multiple decision trees. XGBoost provides the capability to effectively manage extensive datasets, achieve optimal performance in tasks like regression and classification, and effectively handle missing values in real-time data with both rapidity and precision. However, XGBoost, as a tree-based model, has the potential to excessively fit the data, particularly when the trees are excessively deep and the data contains noise. The training process for the decision trees in this model is sequential, where the outcome of the current tree influences the construction of the next tree [18]. XGBoost is recognized as one of the best-performing decision-tree-based models due to its ability to fine-tune numerous hyperparameters to enhance performance. Key hyperparameters include n_estimators, which represents the number of decision trees, and max_depth, which defines the maximum depth of a decision tree [19]. Additionally, L1 and L2 regularization parameters apply penalties to feature weights to prevent overfitting, among other parameters.

Meanwhile, the ANN and RNN models are two deep learning models developed in this study. These models can learn more complex data relationships compared to the previous three models because they have parameters that can be adjusted to increase their complexity, such as the number of hidden layers, the number of nodes per layer, activation functions for each layer, learning rate, optimizer, epochs, and batch size [3]. The main difference between the ANN and RNN models lies in the RNN’s superior ability to learn from sequential input [20]. This is due to each node in an RNN acting as a memory cell, which also increases the complexity of the RNN model. In addition to setting parameters for each model, this study also varied the character length to determine the optimal number of SMILES characters for predicting the *T_g_* of polymers. However, the main disadvantage of these neural networks models is that they could not perform well if the dataset number was small.

### 2.3. Model Performance Evaluation

In this study, the prediction performance of the model is evaluated using the R^2^ score. The R^2^ score measures how well the model fits the data by assessing the proportion of variance in the dependent variable that is explained by the independent variables. This metric ranges from −∞ to 1, where the model’s accuracy improves as the R^2^ score approaches 1. In general, the quality of prediction by machine learning is said to be good if the R^2^ score is equal to or more than 0.8 [21]. Equation (1) shows the calculation of the R^2^ score, where n represents the number of data points, y is the actual output value, $\bar{y}$ is the mean output value, and $\hat{y}$ is the model’s predicted value [22].

(1) $$R^{2}\;score=1-\frac{\sum_{i=1}^{n}{\left({\hat{y}}_{i}-y_{i}\right)}^{2}}{\sum_{i=1}^{n}{\left(y_{i}-\bar{y}\right)}^{2}}$$

In addition to using the R^2^ score, model prediction performance evaluation was also conducted by examining the stability of the model using k-fold validation and the training time required by each model.

## 3. Results

### 3.1. Machine Learning Model Prediction Performance

**Figure 1:**
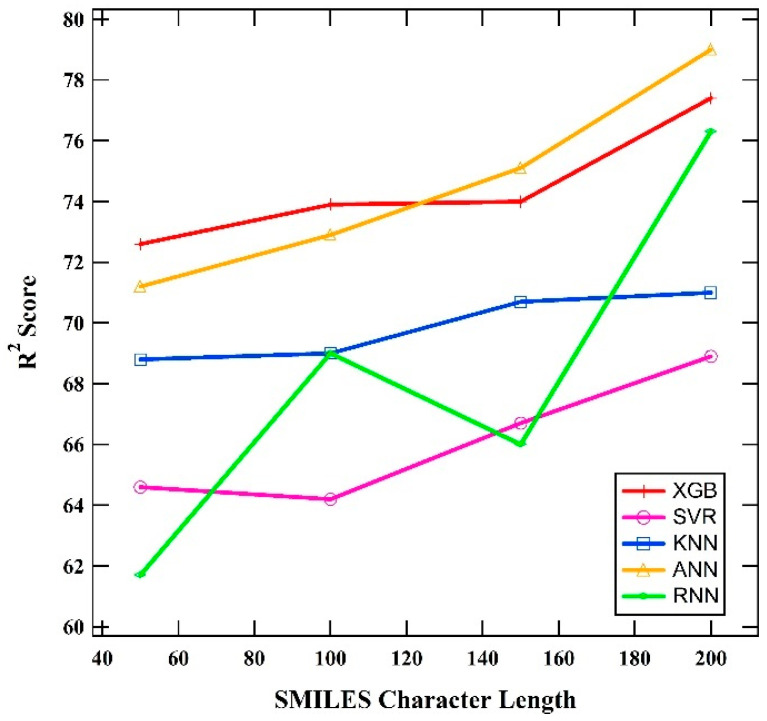
Comparison of the relationship between SMILES character length and model performance.

In this study, the fine-tuning method was used to determine the optimal combination of hyperparameters for each model, where variations in hyperparameter values were established before the model training process. Additionally, the effect of SMILES character length used as input in training was evaluated. Figure 1 shows the impact of SMILES character length on the performance of the five machine learning models, represented by the R^2^ score. Based on the graph, the optimal SMILES character length for use as input in training the machine learning models is 200.

**Figure 2:**
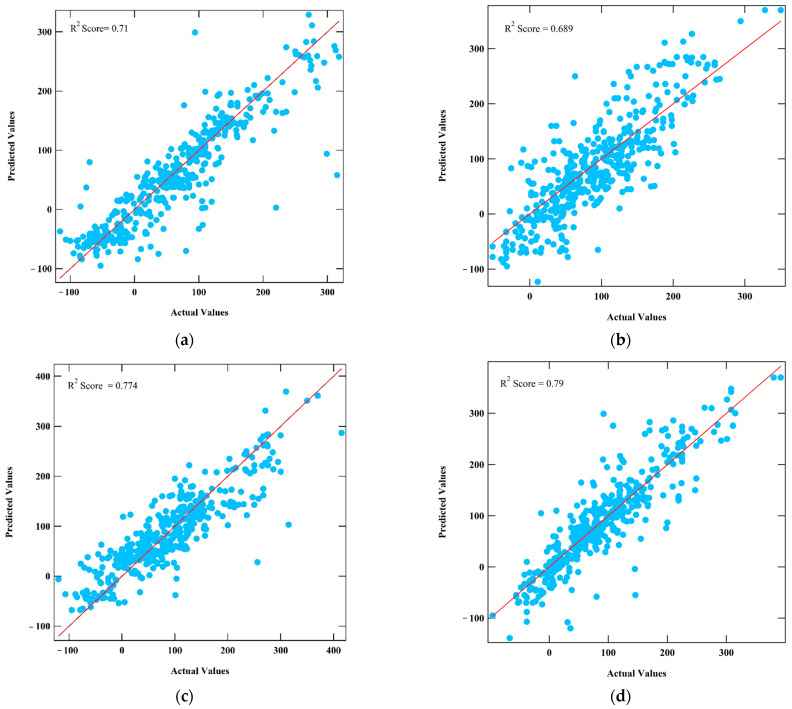

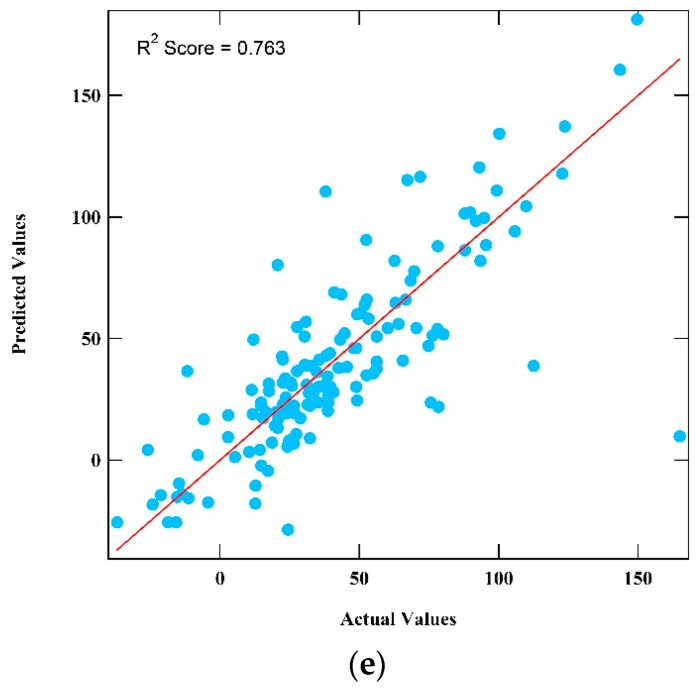
Performance and data distribution of the KNN (**a**), SVR (**b**), XGBoost (**c**), ANN (**d**), and RNN (**e**) models trained with the optimal parameters. The blue dots represent the testing data points, while the red line denotes a condition where the true value is the same as predicted value.

Table 2 summarizes the optimized hyperparameter values to obtain the highest prediction performance for the five machine learning models employed. Figure 2 shows the prediction performance (R^2^ scores) and actual-predicted data distribution of the KNN, SVR, XGBoost, ANN, and RNN models trained using the optimized hyperparameter. The results indicated that the ANN model has the best prediction performance, with an R^2^ score of 0.790, while the model with the lowest performance is the SVR, with an R^2^ score of 0.689.

**Figure 3:**
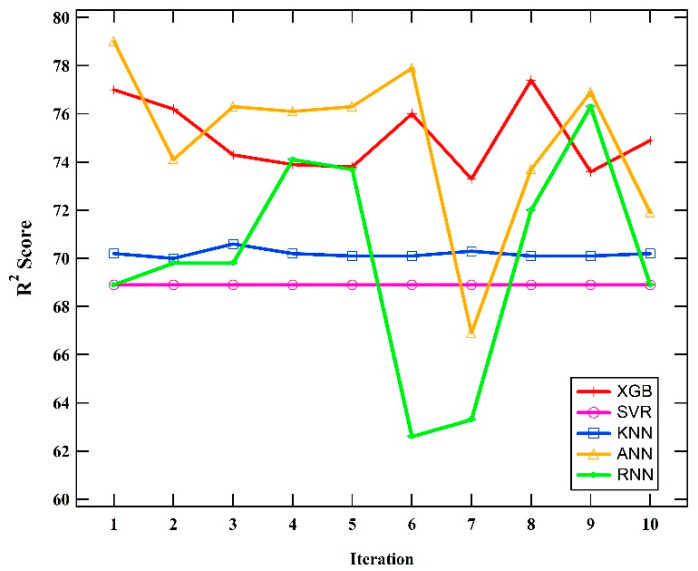
Comparison of model performance stability over 10 iterations using K-fold method.

The k-fold method was used to measure the stability of each model’s performance. This method partitions the dataset into k segments, enabling the use of each segment as testing data. Figure 3 illustrates the k-fold method’s performance stability for the five models using a k value of 10. Based on the graph, the XGBoost model has the highest performance stability compared to the other four models.

In addition, the time needed to train the model could be an issue. For example, it is known that deep neural network methods require more time to train the model than simple machine learning because of the neural network nature of stochastics methods [23]. Table 3 shows that RNN requires much time in comparison to KNN, SVR, XGBoost, and ANN.

Furthermore, the Diebold–Mariano (DM) test was used to determine the significance of the difference in prediction performance. The DM test calculates a test statistic, known as the DM statistic, which quantifies the standardized difference in loss between the two models. A statistically significant deviation from zero in the DM statistic indicates that one model outperforms the other [24]. Table 4 displays the outcomes of the DM test comparing XGBoost, ANN, and RNN. We excluded the KNN and SVR models due to their significantly lower prediction performance compared to the other three models.

### 3.2. Model Validation for Predicting the T_g_ of Polymers Using SMILES Descriptors

The XGBoost model is selected as the primary model for predicting the *T_g_* values of five new polymer compounds outside of the dataset used in model development based on the description of the model performance provided above. This is because of its stable performance and significantly lower training time than other models. Table 5 displays the predicted *T_g_* values for these five novel polymer compounds. The SMILES character * represents the polymerization site.

## 4. Discussion

The results of this study provide valuable insights into the prediction of the glass transition temperature (*T_g_*) of polymers using various machine learning models. The fine-tuning method used to determine optimal parameters highlights the necessity of meticulous parameter optimization to enhance model performance. The evaluation of the SMILES character length reveals that an input length of 200 characters is optimal for model training. This is because SMILES character lengths below 200 do not adequately describe the polymer structure, while lengths greater than 200 result in the curse of dimensionality, which degrades model performance. This finding aligns with previous studies that emphasize the importance of input representation in machine learning models for predicting *T_g_* properties of polymers.

Among the models tested, the ANN model achieved the highest R^2^ score of 0.790, demonstrating its superior ability to capture the complex relationships between SMILES descriptors and *T_g_* values. The prediction performance stability of the model should be taken into account since, for a neural network, given a small number of data points, the results can be fluctuating. It should be noted that even though the R^2^ scores of all models are well below 0.8 and even though it cannot be a good fit, if the purpose is to screen and predict from thousands of polymers, R^2^ scores above 0.75 can be used with care. The XGB model’s nearly equivalent performance (R^2^ score of 0.774), combined with its shorter training time and higher stability, makes it a more practical choice for large-scale applications. In contrast, the lower performance of the SVR model indicates that it is less suitable for this prediction task due to its sensitivity to parameter settings and the characteristics of SMILES data. It is unsurprising that XGBoost demonstrated the best prediction performance in terms of stability, despite ANN providing the maximum prediction performance; however, the prediction performance fluctuates, as shown in Figure 3. According to the ten-time rule, the number of data points should be ten times more than the number of variables in predictive regression [25]. Therefore, with only 1437 data points in this paper, we can classify our data set as small. XGBoost and other simple machine learning models, such as KNN and SVR, are known as preferred models for small datasets. However, simple machine learning models sometimes cannot capture the complexity relation between input and output features. However, XGBoost could outperform ANN in cases with high data dimensions [26], such as our case with 200 features encoded as SMILES binary numbers.

The significant difference in training times between models using the OHE method (KNN, SVR, XGB, ANN) and the NLP method (RNN) highlights the efficiency of the OHE approach for converting SMILES descriptors into numerical data. The reason may be due to the complexity of the architecture, which causes the descriptor to take a long time to process. This efficiency is crucial for practical applications where computational resources and time are limited. Meanwhile, the stability analysis using the k-fold method (k = 10) shows that the XGB model has the highest performance stability among the five models tested. This stability is essential for ensuring reliable predictions across different subsets of data, enhancing the model’s robustness and generalizability. From the results of Diebold–Mariano (DM) test, it can be concluded that XGBoost, ANN, and RNN prediction performance is different enough.

**Figure 4:**
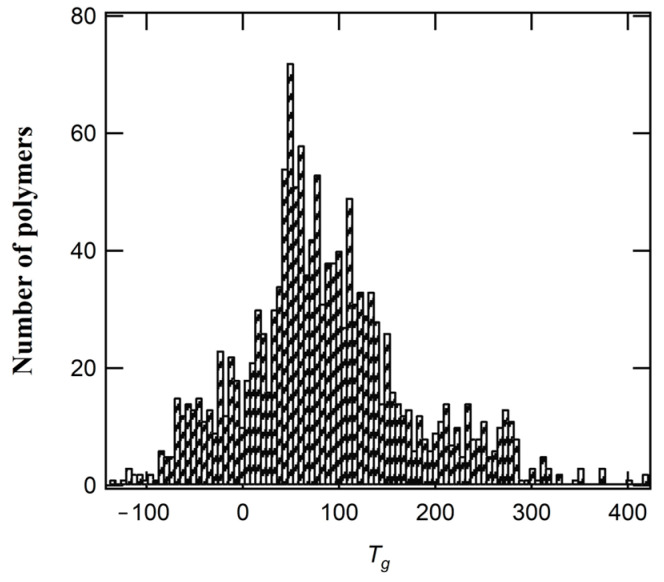
The *T_g_* polymer data distribution.

The XGBoost model is excellent at predicting *T_g_* values of polymers, especially those with *T_g_* < 200 °C. However, when *T_g_* exceeds 200 °C, the prediction loses accuracy and requires caution. Figure 4 illustrates the division of the dataset. The number of polymers that have *T_g_* > 200 °C is considerably low in comparison to those with *T_g_* < 200 °C and thus affects prediction performance. The relatively low deviation also highlights the model’s potential to reduce experimental costs and time associated with determining *T_g_* values in a laboratory setting.

## 5. Conclusions

This study effectively applied simple machine learning and deep learning models to predict the *T_g_* of polymers using SMILES descriptors. Key findings include the importance of SMILES character length, with less than 200 characters failing to describe compound structures accurately and more than 200 characters reducing performance due to the curse of dimensionality. Among the models tested, the ANN model achieved the highest R^2^ score of 0.79, but its performance was still considered relatively low. The XGB model demonstrated the highest stability and reasonable accuracy, with an R^2^ score of 0.774, making it the preferred model due to its shorter training time and robust performance. The OHE method for SMILES conversion proved more efficient than NLP, as shown by faster training times in the KNN, SVR, XGB, and ANN models compared to the RNN model. Validation of new polymer data confirmed the XGB model’s robustness, which can be used for predicting *T_g_* < 200 °C, and beyond that value it should be used carefully. These results underscore the importance of optimizing SMILES descriptor conversion and model parameters to achieve reliable predictions. Future research should focus on improving model accuracy and generalizability by incorporating additional features and advanced techniques. This study also contributes to the development of reliable predictive models for polymer properties, aiding in the design and application of new polymer materials.

## Figures and Tables

**Table 1:** Descriptive statistics of polymer dataset.

Data	Min	Max	Average	Std. Dev.
Character length	3	170	48.22	27.8
*T_g_* value	−139	420	85.40	88.82

**Table 2:** Optimized hyperparameters for ML models based on fine-tuning results.

Model	Parameter	Optimal Value
KNN	Character length	200
	n_neighbors	8
SVR	Character length	200
	Kernel	RBF
	C	1
	gamma	0.01
XGB	Character length	200
	max_depth	10
	learning_rate	0.1509741801833367
	n_estimators	2.095
	min_child_weight	20
	gamma	0.010500376855063191
	reg_lambda	0.007188240690305372
	reg_alpha	2.4700851023872214 × 10^−6^
ANN	Character length	200
	Number of hidden layers	3
	Number of nodes per hidden layer	512, 256, 128
	Activation function for input layer	ReLU
	Activation function for hidden layer	ReLU
	Activation function for output layer	Linear
	Optimizer	Adam
	Loss function	MSE
	Epoch	100
	Batch size	479
	Learning rate	0.0001
RNN	Input dim	45
	Input len	200
	Activation function for input layer	ReLU
	Activation function for hidden layer	ReLU
	Activation function for output layer	Linear
	Optimizer	Adam
	Loss function	MSE
	Epoch	500
	Batch size	479
	Patience	50

**Table 3:** Training time for each model using optimal parameters.

Model	Training Time
KNN	4 s
SVR	18 s
XGB	7 s
ANN	30 s
RNN	14 h 12 min

**Table 4:** The Diebold–Mariano test comparison for XGBoost, ANN, and RNN.

Model	Diebold–Mariano Test Statistic Value	*p*-Value
XGBoost-ANN	1.12	0.78
XGBoost-RNN	1.56	0.27
ANN-RNN	0.48	0.45

**Table 5:** Predicted *T_g_* results for polymer compounds outside the dataset.

Polymer Compounds	SMILES	*T_g_* Actual	*T_g_* Predicted	Delta
poly(ethyl 2-fluoroacrylate)	CCOC(=O)C(C*)(F)*	94	97.3	3.3
poly[(phenylarsandiyl)(1-phenylethene-1,2-diyl)]	*C=C([As](c1ccccc1)*)c1ccccc1	92.9	97	4.1
poly{1-[(2,2-difluoroethane-1,1,2-triyl-1-oxy)methoxy]-2,2-difluoroethylene}	*C(C(F)(F)*)OCOC(C(F)(F)*)*	122	122.7	0.7
poly(2-phenylacetate)	*CC(C(=O)c1ccc(cc1)C)*	71	69.5	1.5
poly[(4,4′-methylenedianiline)-alt-(terephthaloyl dichloride)]	*Nc1ccc(cc1)Cc1ccc(cc1)NC(=O)c1ccc(cc1)C(=O)*	300	260.8	39.2

## Data Availability

Data used in this study can be accessed at https://github.com/PolymerTg/Polymer-Tg-Machine-Learning, accessed on 1 August 2024.
